# Myeloid Disease with the CSF3R T618I Mutation after CLL

**DOI:** 10.1155/2020/6670965

**Published:** 2020-12-21

**Authors:** Maria Eduarda Couto, Susana Bizarro, Domingos Sousa, Nelson Domingues, Isabel Oliveira, Gabriela Martins, Manuel R. Teixeira, Mário Mariz

**Affiliations:** ^1^Onco-hematology Department, Instituto Português de Oncologia Do Porto F.G. E.P.E., Porto, Portugal; ^2^Genetics Department, Instituto Português de Oncologia Do Porto F.G. E.P.E., Porto, Portugal; ^3^Internal Medicine Department, Centro Hospitalar e Universitário Do Algarve E.P.E., Porto, Portugal; ^4^Immunology Department, Instituto Português de Oncologia Do Porto F.G. E.P.E., Porto, Portugal; ^5^Biomedical Sciences Institute, University of Porto, Porto, Portugal

## Abstract

Chronic lymphocytic leukemia (CLL) is frequently an indolent diagnosis, with most of the patients being under surveillance for long time. There is an increased risk of a second neoplasia in CLL, rarely hematological (in the myeloid lineage is even rarer). A 58-year-old male was diagnosed with CLL in 2012, remaining in regular surveillance until 2014. Then, the CLL progressed, and 6 cycles of rituximab, fludarabine, and cyclophosphamide were prescribed with partial response. He remained in surveillance and suffered 2 episodes of autoimmune hemolytic anemia until 2019. Then, the hemolytic anemia relapsed and a neutrophilia became evident (progressing slowly), as well as a thrombocytopenia and splenomegaly without adenopathy were found. The bone marrow aspirate showed a chronic myeloproliferative disease without dysplasia. A peripheral blood search for the *CSF3R* mutation (T618I) was positive, also suggesting Chronic Neutrophilic Leukemia (CNL). For a discrete monocytosis, a chronic myelomonocytic leukemia (CMML) was also considered. Hydroxyurea was then prescribed. The T618I CSF3R mutation is highly suggestive of CNL (being diagnostic criteria for CNL); however, this case may also suggest CMML as a possible diagnosis (there are other mutations in the CSF3R gene described for CMML, but not the T618I, which is highly exclusive of CNL according to the literature). To our knowledge, this is the first report of a possible CNL in a CLL patient (the opposite was already described in 1998).

## 1. Introduction

Chronic lymphocytic leukemia (CLL) is a very common and usually indolent lymphoid malignancy. Most patients remain in surveillance for a long time. CLL increases the risk of secondary malignancies, mainly in the skin and digestive tract, by diminishing the humoral and cellular responses of the immune system.

A Chronic Neutrophilic Leukemia (CNL) is defined by persistent neutrophilic proliferation (mainly mature forms), hyperplasia of the bone marrow granulocyte lineage, and usually splenomegaly. [1] It is a BCR-ABL negative myeloproliferative neoplasm, named in 1964 for the first time. It is a rare event typically found in elderly people, more frequently males [1]. Patients are usually asymptomatic, but there is a hemorrhagic tendency probably caused by the neutrophilia and vascular infiltration by the disease [1]. The recent finding of the activating mutations of the gene *CSF3R*, the T618I (found in 80% of the cases), revolutionized the understanding of the pathogenesis of this disease, nowadays being an important diagnosis criterion [1–6]. However, CNL still is an exclusion diagnosis.

The diagnosis of a chronic myeloid neoplasia as CNL in a patient with a chronic lymphoid neoplasia is a rare phenomenon, mainly when the first diagnosis is CLL [7–8]. As far as we know, there is only one case report describing a CLL after a CNL, published in 1998 [7].

## 2. Case Report

A 58-year-old male was diagnosed with CLL Rai 1 in 2012, remaining in regular surveillance. He had a past medical history of hyperuricemia, hypertension, hypothyroidism, prostate benign hyperplasia, biliary calculus, and clear cells carcinoma of the right kidney (submitted to partial nephrectomy).

In 2014, the CLL progressed with anemia, growing adenopathy, and fast doubling of the leucocyte count. Rituximab, fludarabine, and cyclophosphamide were prescribed for 6 cycles, and the patient achieved a partial response, remaining in surveillance afterwards.

One year later, he was hospitalized for febrile neutropenia caused by right base pneumonia, in septic shock and multiorganic failure (being admitted in the ICU). This episode was followed by another admittance for febrile neutropenia with respiratory symptoms in 6 months.

In 2016, the patient developed autoimmune hemolytic anemia. Prednisolone 1 mg/kg and folic acid were prescribed, being stopped in 3 months with a fast relapse of the hemolysis. A second treatment course was performed for 7 months, with stabilization of the hemoglobin levels around 11-12 g/dL.

Suddenly in the end of 2018, there was a significant increase in the leucocyte count, mainly caused by a neutrophilia (Table 1). The previous kidney cancer had no signs of relapse, and there was no infectious process in the course. There was a soft decrease in the hemoglobin level, but the increase in the leucocyte and neutrophil counts persisted, with the identification of immature myeloid forms in the peripheral blood smear and 0.31 × 10⁹/L of blast cells. A leukemic reaction was suspected. In three months, the hemolytic anemia relapsed, preceded by a dental abscess. The patient complained about asthenia, loss of weight (5 kg in 5 months), bone pain, and nausea. He denied other symptoms. The leukocytosis and neutrophilia became even more evident, as well as the thrombocytopenia. The direct coombs test was positive for complement and IgG (1 : 30). An abdominal echography showed homogeneous splenomegaly (15.8 cm). The flow cytometer of the peripheral blood showed polyclonal B lymphocytes circulating. The bone marrow aspirate was morphologically suggestive of a chronic myeloproliferative disease without dysplasia. The del17p was found by FISH (fluorescence in situ hybridization) in the bone marrow aspirate. The flow cytometer of the bone marrow aspirate identified a major population of the neutrophilic lineage in different stages of maturation, without blasts; no signs of the CLL were found at this point. One month later, a second bone marrow aspirate showed the same morphological results; the karyotype analysis of the bone marrow aspirate identified an isochromosome 17q. Prednisolone was restarted 1 mg/kg with improvement of the anemia but worsening of the thrombocytopenia (to values inferior to 100 × 10⁹/L). The search for the *CSF3R* gene mutations in the peripheral blood was positive for the most common mutation (T618I), which is a defining criterion for CNL. After 3 months, the patient became clinically asymptomatic with reduction of the leucocyte count and splenomegaly with hydroxyurea 5 gr/week, also under prednisolone 20 mg daily for the hemolytic anemia. After 12 months, the leukocytosis with neutrophilia increased, blasts were identified in the peripheral blood, and a splenomegaly became evident. The patient was not eligible for bone marrow transplant, becoming a palliative case. Multiple nodules were found in a cerebral CT scan (requested for neurologic complains), possibly due to kidney cancer relapse, which were never biopsied for the severe thrombocytopenia.

## 3. Discussion

It is well known that CLL is a disease that remains in constant clonal evolution [9]. The CLL is commonly associated with an increased risk of a second neoplasia, but rarely hematologic (and even rarer a myeloid event) as we report here. As far as we know, there is only one similar case, in which the CNL preceded the CLL diagnosis, in an era when genomics were not part of the diagnosis criteria for CNL [7]. Besides being rare, CNL must be considered in this clinical case, as well as a CMML.

The clinic signs, blood counts, bone marrow results, and molecular biology studies were highly suggestive of a myeloproliferative malignancy. The thrombocytopenia could be explained by the increasing spleen, often heralding a blast crisis. LDH and B12 vitamin levels are typically high in a leukemoid reaction, with low levels of G-CSF [1].

There are many findings suggestive of CNL in this clinical case: a high and persistent neutrophilia without any other explanation for happening; a nearly normal monocyte count (an increase in leucocytes could have caused an increase in the absolute value of monocytes); the bone marrow studies (conducted in two different occasions) evidencing a myeloproliferative process; and the flow cytometry identifying a major population of the neutrophilic lineage in different stages of maturation, without blasts. The search for the activating mutation T618I of the CSF3R gene is essential for the final diagnosis of CNL. This gene is related to other disorders apart from CNL, such as CMML, severe congenital neutropenia, hereditary chronic neutrophilia, and rarely myeloid leukemia, and recently, it was described in the literature also in atypical CML [10]. However, the specific T168I mutation was never described in CMML [11]. CNL is not associated with lymphoproliferative neoplasms other than CLL (there is only one case report of a CLL after a CNL, but not the opposite) [7].

CMML should be considered too. Apart from the neutrophilia, this patient also had a monocyte count in the upper limit of normal values (absolute counts regularly between 0.94 and 1,94 × 10^9^/L, having one episode with 0.39 × 10^9^/L), a persistent monocytosis. CMML is a more common diagnosis compared with CNL.

The loss of the short arm of chromosome 17 (where the TP53 gene is located) identified in the bone marrow aspirate is a genetic abnormality typically found in CLL (as well as the hemolytic events) but not in myeloproliferative diseases. However, there were no signs of CLL in the flow cytometry at the myeloid diagnosis time. The archival cytogenetic sample did not have sufficient quality to allow the study for the CSF3R mutation or p53 mutation.

In what refers to the therapeutic approach, a cytoreduction with hydroxyurea is usually successful in the leukocytosis control and splenomegaly (25% of the patients are refractory) for both CNL and CMML [3, 11].

The main therapeutic options for CNL are interferon, hypometlylating agents, ruxolitinib, thalidomide, and cladribine [3]. TKI therapy was experimented successfully in a CNL patient for the first time in 2004, for the CSF3R mutation [12]. Treatment with the SRC kinase inhibitor dasatinib and the JAK1/2 kinase inhibitor ruxolitinib also have shown efficacy for CNL with membrane proximal mutations and truncation mutations, respectively [3, 12, 13]. Most of the agents proposed for CNL treatment are palliative, not achieving remission of the disease. There is little role for bone marrow transplantation (the only curative option) or induction regimens, given the bad results obtained [1]. The prognosis of CNL is not favorable nowadays: between 20–30 months of median survival and a transformation rate to AML between 10 and 21. 2% in a median time of 21 months. Due to the rarity of the disease, there is no prognostic scoring system approved to guide treatment options and follow-up [1, 3, 8].

The main treatment options for CMML are hypomethylating agents (such as 5-azacitidine or decitabine), with an overall response rate of ∼30–40% and complete remission rate of ∼7–17%, with no impact on mutational allele burdens. Allogeneic stem cell transplant is the only potentially curative option, but it is associated with significant morbidity and mortality [11].

The authors emphasize the importance of the T618I mutation in the CNL diagnosis, and they wonder whether it is enough to exclude the CMML diagnosis. A wider targeted genotyping could be appropriate to better clarify the etiology of this disease. Future investigations should address the T618I mutation presence in a broad group of CMML patients, in order to strengthen its specificity. Furthermore, the impact of this mutation in selecting treatment options for CNL should also be better understood.

## Figures and Tables

**Table 1:** Blood counts obtained in a temporal line (from the left to the right; time 0 is the moment of the myeloid diagnosis and the beginning of hydroxyurea).

	17 months before	16 months before	12 months before	7 months before	5 months before	3 months before	2 months before	1 month before	Time 0 (HU started)	1 month later	2 months later	3 months later	4 months later	6 months later	12 months later
Hemoglobin g/dl	10.7	11.6	12.1	11.4	11.4	9.4	8.9	11.3	11.5	9,5	12,4	11,1	11,4	9,4	8,2
Leucocytes × 10^9^/L	9.96	8.33	11.59	13.81	24.82	62.71	77.8	81.7	84.01	21,15	38,61	14,91	61,48	71,77	117,01
Neutrophils × 10^9^/L	5.94	5.03	7.74	10.21	19.94	51.23	63.8	69.48	72.67	15,69	34,77	13,08	53,96	63,02	94,78
Eosinophils × 10^9^/L	0.34	0.18	0.20.18	0.14	0.22	0.63	0.78	0.03	0	0,03	0.02	0	0,03	0	0
Basophils × 10^9^/L	0.05	0.05	0.1	0.07	0.18	0.63	0	0.05	0.34	0,08	0,12	0,04	0,03	0	0,59
Lymphocytes × 10^9^/L	2.3	2	2.24	2.09	1.96	2.76	3.11	2.01	1.01	1,11	1,96	1,11	1,98	6,48	2,34
Monocytes × 10^9^/L	1.23	1.02	1.07	1.12	1.23	0.94	0.39	1.94	1.09	2,65	0,53	0,45	1,03	1,09	9,95
Immature granulocytes × 10^9^/L	0.1	0.05	0.16	0.18	1.29	Metamielocytes 2,76 × 10^9^/L, mielocytes 2,20 × 10^9^/L, promielocytes 1,25 × 10^9^/L	Metamielocytes 5,45 × 10^9^/L, mielocytes 4,28 × 10^9^/L	8.19	Metamielocytes 2,86 × 10^9^/L, mielocytes 4,62 × 10^9^/L, promielocytes 1,43 × 10^9^/L	1,59	1,21	0,23	4,45	Metamielocytes 1,82 × 10^9^/L, mielocytes 0,36 × 10^9^/L	Metamielocytes 4,1 × 10^9^/L, mielocytes 1,76 × 10^9^/L, promielocytes 1,76 × 10^9^/L, blasts 1,76 × 10^9^/L
Platelets × 10^9^/L	263	189	228	211	193	204	147	117	96	77	50	66	67	86	14
Blasts × 10^9^/L						0.31									
Sedimentation velocity							20/45								
